# Effects of environment and genotype on dispersal differ across departure, transfer and settlement in a butterfly metapopulation

**DOI:** 10.1098/rspb.2022.0322

**Published:** 2022-06-08

**Authors:** Michelle F. DiLeo, Etsuko Nonaka, Arild Husby, Marjo Saastamoinen

**Affiliations:** ^1^ Research Centre for Ecological Change, Organismal and Evolutionary Biology Research Programme, Faculty of Biological and Environmental Sciences, University of Helsinki, Helsinki, Finland; ^2^ Ontario Ministry of Northern Development, Mines, Natural Resources and Forestry, Peterborough, ON, Canada; ^3^ Department of Biological and Environmental Sciences, University of Jyväskylä, Jyväskylä, Finland; ^4^ Evolutionary Biology, Department of Ecology and Genetics, Uppsala University, Uppsala, Sweden; ^5^ Helsinki Institute of Life Science, University of Helsinki, Helsinki, Finland

**Keywords:** dispersal, genetic assignment tests, genotype-by-environment interactions, butterfly, patch quality, fitness

## Abstract

Active dispersal is driven by extrinsic and intrinsic factors at the three stages of departure, transfer and settlement. Most empirical studies capture only one stage of this complex process, and knowledge of how much can be generalized from one stage to another remains unknown. Here we use genetic assignment tests to reconstruct dispersal across 5 years and 232 habitat patches of a Glanville fritillary butterfly (*Melitaea cinxia*) metapopulation. We link individual dispersal events to weather, landscape structure, size and quality of habitat patches, and individual genotype to identify the factors that influence the three stages of dispersal and post-settlement survival. We found that nearly all tested factors strongly affected departure probabilities, but that the same factors explained very little variation in realized dispersal distances. Surprisingly, we found no effect of dispersal distance on post-settlement survival. Rather, survival was influenced by weather conditions, quality of the natal habitat patch, and a strong interaction between genotype and occupancy status of the settled habitat patch, with more mobile genotypes having higher survival as colonists rather than as immigrants. Our work highlights the multi-causality of dispersal and that some dispersal costs can only be understood by considering extrinsic and intrinsic factors and their interaction across the entire dispersal process.

## 1. Introduction

It is now well recognized that active dispersal is complex, involving individual decisions at three connected stages of departure, transfer and settlement [1–3]. Yet dispersal is often modelled in simplified ways, e.g. collapsing the three stages into one or averaging over individual differences with fixed emigration probabilities or dispersal kernels [3,4]. This has prompted calls for ecologists to adopt more realistic views of dispersal into models of connectivity [5], invasions [6], range dynamics [7,8], and responses to climate change [9,10]. However, the question of how much dispersal realism is needed to accurately model these outcomes remains unclear [9]. This stems partly from a lack of understanding of how the complexity of dispersal plays out in real populations. The inherent challenges of tracking dispersal in the wild means that our empirical knowledge are often piecewise, coming from observations of only one stage of the dispersal process and under a limited set of environmental conditions [11]. There is thus a critical need for empirical studies that follow individuals through all three stages of dispersal, investigating multiple drivers and on scales relevant for predicting population dynamics.

Individual decisions to leave natal habitat, how far to travel, and where to settle, depend on both extrinsic (e.g. environmental) and intrinsic (e.g. condition and genotype) factors [2]. While there have been attempts to draw generalities about the causes of dispersal across species [12–14], few have tested if causes are consistent across dispersal stages (but see [15–17]). Positive correlation of emigration rates and dispersal distance are often assumed [18], and there is empirical evidence for this—experiments have found that individuals are more likely to both leave and disperse further from poor-quality natal sites (e.g. [19,20] Likewise, positive correlations among stages can arise when individuals vary in traits that affect their quality as a disperser (e.g. silver spoon effects, [21]). However, stages of dispersal may be decoupled if each involves different costs and constraints that are realized over different spatial scales [22,23]. Extrinsic cues that trigger departure (e.g. the presence of kin or poor-quality habitat) are perceived at the scale of the natal habitat, and the act of departure itself may not be that costly (although departure might carry other ecological costs such as those related to missed opportunities in natal habitat patch) [1,22]. By contrast, movement through the matrix is experienced over larger spatial scales and carries a high physiological cost and predation risk [22]. Consequently, extrinsic factors such as habitat patch quality are expected to strongly affect departure and settlement, whereas landscape structure is expected to more strongly constrain the transfer stage [1]. It follows that intrinsic dispersal ability should also disproportionately affect the transfer stage; intrinsic differences often have a strong genetic basis [24] involving a correlated suite of morphological, physiological or behavioural traits (i.e. dispersal syndromes; [25]). Growing evidence suggests that these syndromes do not necessarily divide individuals into ‘good’ or ‘poor’ dispersers but translate to individual differences in perceptual ability [26], personality [27], habitat preferences [28] or thermal optima [29]. This can lead to complex outcomes at each of the three stages of dispersal, especially if intrinsic differences interact with extrinsic factors (e.g. genotype-by-environment interactions; [30–33]). However, these interactions are rarely considered in field studies (but see [34–36]) and to our knowledge have not been tracked through distinct dispersal stages.

Here we use genetic assignment tests to reconstruct the three stages of dispersal across a Glanville fritillary butterfly (*Melitaea cinxia*) metapopulation in the Åland Islands, Finland. Capitalizing on extensive ecological data and a known polymorphism in the flight-related *phosphoglucose isomerase* (Pgi) gene [37], we link individual dispersal events to environment and individual genotype to ask: (i) what are the contributions of environment, genotype, and genotype-by-environment interactions (G x E) to realized dispersal? (ii) Are driving factors consistent in their effects across the stages of departure, transfer and settlement? (iii) How do factors at each stage impact post-settlement survival? Previous work on the species identified a genotype-by-temperature interaction in flight distance [29,38]; however, how this translates to realized dispersal in the field and where it fits in the context of other ecological drivers of dispersal remains unknown. By quantifying effects of dispersal on post-settlement survival, our study further resolves the stages at which costs of dispersal are incurred. These deferred costs are rarely tracked but are crucially important for predicting population responses in changing environments [22].

## 2. Methods

### (a) Study system and sample collection

**Figure 1.**
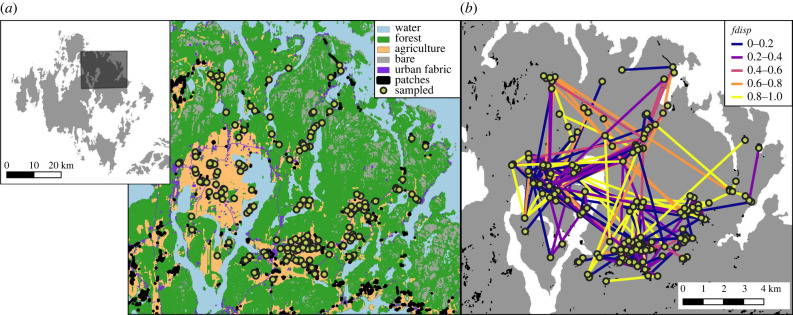
Map of study area showing generalized landcover (*a*) and reconstructed dispersal paths from assignment tests (*b*). The left inset highlights the study area in relation to mainland Åland. Points show sampled patches and the colour of lines in (*b*) reflect the assigned family's frequency of the mobile genotype (*fdisp*). (Online version in colour.)

The Glanville fritillary butterfly (*Melitaea cinxia*) is a small butterfly in the checkerspot family with a broad Eurasian distribution. At its northern range limit, the species occurs in the Åland Islands, Finland, where it occupies a metapopulation network of over 4000 habitat patches. Habitat patches (hereinafter ‘patches’) are a mix of dry meadows, pastures, roadsides and rocky outcrops with well-defined boundaries, and contain one or both of the butterflies’ host plants, *Plantago lanceolata* and *Veronica spicata*. The metapopulation is highly dynamic with frequent local extinctions (Ovaskainen and Saastamoinen [39]). Dispersal leading to recolonization is thus highly important for the maintenance of the metapopulation. Butterflies mate in their natal patch shortly after eclosion in early summer and females then oviposit in their natal patch and/or disperse to a new patch [40]. Every meadow (i.e. potential habitat for a local population) in the metapopulation has been visited each autumn since 1993 to census larval nests, which are conspicuously woven by larval groups at the base of their host plants [40]. From 2007 to 2012, three larvae were collected from every nest found in the region of Saltvik, a 10 × 10 km area in the northeast of Åland (figure 1). DNA was extracted from 10 000 larvae and samples were genotyped at 272 SNPs as described in Fountain *et al.* [41,42]. In brief, the panel of SNPs is a mix of putatively neutral markers located in non-coding regions of the genome (*n* = 40), markers from genes that were differentially expressed in experimental flight treatments or previously related to flight ability (*n* = 188; [43–46]), and 44 markers selected to cover the remaining chromosomes not represented by the other markers [41,42]. We retained 245 SNPs that passed quality control and had individual and SNP call rates greater than 95%.

### (b) Assignment tests

A previous study reconstructed a limited number of dispersal events from cases where females oviposited and had surviving nests in multiple meadows but was unable to identify natal patches [42]. To identify putative natal patches, we used the genetic assignment program GENECLASS2 [47] to assign full-sib larval families identified in a previous paper [42] to patches sampled in the previous year. Most sampled nests consist of a single full-sib family, but in the minority of cases full-sibs were found in multiple nests (e.g. due to nest splitting or a female laying several clutches) or a single nest consisted of multiple families due to nest merging. Accordingly, sample sizes were on average three larvae per full-sib family but ranged from 1 to 20. We used the Bayesian Rannala and Mountain method [48] for assignment, which computes the likelihood that a group of individuals originates from a given reference population. An assignment score is then calculated for each reference population reflecting how well the sample fits [47]. The score of group *i* in population *T* ranges from 0 to 100 and is computed as

$${\mathrm{score}}_{i,T}=\;\frac{L_{i,T}}{\sum_{\;j=1}^{P}L_{\;j,T}},$$

where *P* is the number of reference populations (i.e. source populations) and *L_i,T_* is the likelihood of group *i* in population *T* [47]. Based on results of simulations (see below), we only considered samples with scores equal to 100 as true assignments. In cases where full-sibs were found in multiple patches, we split the family by patch before assignment.

Genetic assignments were then transformed into estimates of dispersal. Departure probabilities were modelled as a binomial response, with a value of 1 if the family was assigned to a different patch than it was sampled in and a 0 if the family was assigned to the same patch it was sampled in. Dispersal distance was measured as the Euclidean distance between sampled and assigned patches. Further details of models are found in the section ‘Modelling dispersal’.

To test if there were any obvious biases in the assignment of families to source patches, we looked for differences in assigned and unassigned samples for key factors including sample size, number of nests in the source patch and source patch genetic differentiation (see electronic supplementary material for the full description).

### (c) Simulations

GENECLASS2 has previously been found to perform well even at levels of genetic differentiation as low as *F*_ST_ = 0.04 [49–51]. However, given the dynamic nature of the metapopulation, we used simulations to validate that GENECLASS2 gives accurate assignments under complex population structures. We used the forward individual-based simulation model developed for the *M. cinxia* metapopulations in the Åland Islands by Nonaka *et al*. [52]. The model incorporates known behaviour, ecology and genetics of the species and is parameterized with published estimates and the long-term survey data from 2001to 2016 (see the electronic supplementary material and [52] for the full description, the model fitting procedure and the code). We subsampled the resulting simulated genotypes to reflect our empirical data as much as possible. Specifically, we removed genotypes of parents from the reference sample, randomly sampled a maximum of three genotypes per full-sib family for both reference and assignment samples, and randomly selected 245 SNPs. To determine the effect of missing patches or families in the reference sample, we ran assignment tests with 100%, 50% and 25% of the simulated reference sample included. We believe that our empirical sample most closely reflects the 50% situation, because although we exhaustively sampled all nests found in the field, within-patch nest detection rates are estimated between 50 and 60% [40]. For each of the three reference samples, we calculated type 1 error rates (i.e. proportion of families assigned to the wrong source patch) and power (i.e. the proportion of assigned families). We tested whether the probability of making an assignment was less likely and the probability of making a misassignment was more likely, if the true source patch showed low genetic differentiation (*F*_ST_) from its nearest neighbour using generalized linear models with binomial error distribution.

### (d) Covariates

**Figure 2.**
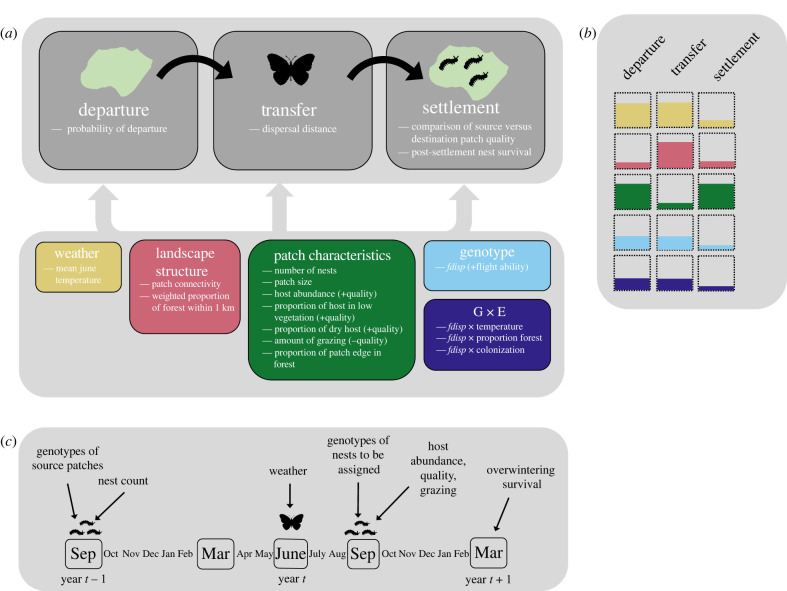
Overview of the study; (*a*) summarizes response variables measured at the three stages of dispersal and the covariates included in the models. The direction of the expected relationship with the response variable is indicated in brackets. (*b*) It shows expected contributions of factors to each of the three stages of dispersal, adapted from [1]. (*c*) It shows a timeline of data collection. Genetic sampling and measurement of patch characteristics took place during autumn surveys in September, overwinter survival was measured during spring surveys in March, and dispersal occurs in June. In all models, patch characteristics of source and destinations were taken from year *t* to be most representative of the conditions experienced by dispersing butterflies. Nest counts of source and destination patches were taken in year *t* − 1 as these are highly correlated with nest counts in the following March, but March data were available only for a subset of years. (Online version in colour.)

We measured several environmental variables associated with source and destination patches to test for their effects on realized dispersal and post-settlement survival (figure 2). These variables included ambient temperature during the flight period and patch characteristics including patch size, nest count and per cent of the patch edge in forest (to account for boundary-crossing effects on dispersal). Patch quality was represented by four variables: host plant abundance, amount of host growing in low vegetation and amount of dry host, which are all positively related to patch quality, and grazing per cent which is negatively to patch quality [53,54]. To capture effects of landscape structure and matrix harshness, we included a measure of patch connectivity and a measure of the distance-weighted proportion of forest within a 1 km buffer surrounding patches (reflecting average dispersal distance from previous work [42]) from Schulz *et al*. [53]. Full details of covariates are in the electronic supplementary material.

To test for the effects of genotype on realized dispersal, we used the candidate locus *phosphoglucose isomerase* (*Pgi*), which is a large-effect locus previously linked to flight ability (reviewed in [37]). Females with the genotype AC or CC have higher flight metabolic rate and move further in the field compared to females with an AA genotype [38,55]. For each family in year *t*, we calculated the combined frequency of the AC and CC genotypes as a measure of the potential flight ability of the parents (hereinafter *fdisp*).

### (e) Controlling for background population structure

To ensure that any detected genetic effects are driven by variation in the *Pgi* locus rather than genetic structure, we included a genetic relationship matrix in downstream models. The pairwise kinship matrix was calculated from a single randomly selected individual per full-sib nest using the VanRaden method in the AGHmatrix package [56,57]. The resulting kinship matrix was included as a random effect in the statistical models (described in the sections below) using either relmatGlmer or relmatLmer functions from the rlme4qtl package [58].

### (f) Modelling dispersal

We fit a generalized linear mixed model to test the effects of weather, landscape structure, source patch characteristics, genotype and genotype-by-environment interactions on the probability of departure using the rlme4qtl package [58]. Departure probability was modelled as a binomial response with a logit-link (1 if the family was assigned to a different patch than it was sampled in, and a 0 if the family was assigned to the same patch it was sampled in). Fixed covariates included those described above, measured for source patches. Nest count and patch area were log-transformed before inclusion in the model to linearize relationships. We further included two-way interactions between the frequency of the dispersive allele and both June temperature and the distance-weighted per cent of forested landscape in a 1 km buffer, as previous work suggests that the genotype with better flight ability flies best in colder conditions [29] and might be more likely to disperse in heavily forested landscapes [59]. We included a random effect of source patch to account for multiple departure events from the same source. For those families that were assigned to departure events, we used a linear mixed effect model implemented in the rlme4qtl package [58] to test the effects of the above covariates on log-transformed dispersal distance.

To test if dispersers tended to successfully settle in patches of similar or higher quality than their source patch, we compared the means of source and destination patch variables using paired *t*-tests, or Wilcoxon signed-rank tests if differences were not normally distributed. We added 1 to nest count before taking the log, as many destination patches had nest counts of 0 (i.e. they were colonized by the disperser). We additionally tested for differences in nest count of source and destination patches excluding patches that were colonized.

### (g) Modelling post-settlement survival

From 2009 to 2012, each larval nest that was sampled in the autumn was re-visited in the spring to quantify overwintering survival (for details see [40]). We tested the effects of weather, landscape structure, patch characteristics, genotype and genotype-by-environment interactions on survival of nests assigned to dispersal events using a generalized liner mixed effects model. Nest survival was fit as a binomial response (0 = dead, 1 = alive) with a logit-link. Fixed covariates included those described above for both source and destination patches, and the log of dispersal distance. We additionally included a two-way interaction between the frequency of the dispersive allele (*fdisp*) and a binary variable describing the colonization status of the target patch (0 = existing population in patch, 1 = colonized patch). We included a random intercept for year to capture broad environmental effects on survival not included in the fixed covariates, and a random intercept for full-sib family to account for multiple nests from the same mother. As the survival data were measured at the nest-level and not the family-level, we excluded nests that were previously determined to consist of non-full-sibs [42]. We additionally ran the models for residents, excluding destination patch characteristics, and tested if residents had higher survival probabilities than dispersers in a separate binomial model.

## 3. Results

### (a) Assignment tests

Assignment tests on simulated data showed low type 1 error rates and moderate power, with assignment probability increasing and misassignment probability decreasing with increasing genetic differentiation between source patch and nearest-neighbour patch (electronic supplementary material, figure S1). Type 1 error and power decreased with assignment score, with a notable sharp decline in error between scores of 99.5 to 100 (electronic supplementary material, figure S1). We thus chose a score of 100 as a safe threshold for retaining assignments for our empirical data. Simulated assignments had type 1 error rates of 0.037, 0.045 and 0.07 for reference populations that were 100%, 50% or 25% subsampled, respectively. The proportion of simulated families that were assigned with a score of 100 were 0.54, 0.46 and 0.34 for reference populations that were 100%, 50% or 25% subsampled, respectively.

In total, 32% (*n =* 799) of our empirical families were assigned to source patches (i.e. had an assignment score of 100). The proportion of assigned families varied per year, from a low of 20% in 2009 to a high of 44% in 2010. Assigned families had significantly higher larval sample sizes (i.e. more larvae genotyped per full-sib family) than unassigned samples, (mean_[assigned]_ = 3.1, mean_[unassigned]_ = 2.4; likelihood ratio test on Poisson regression: *χ*^2^ = 109.4, *p* < 0.001). Assigned families came from source patches with a significantly higher number of larval nests than what was expected from bootstrapping nest counts of available source patches per year (mean_[observed]_ = 11.0, mean_[expected]_ = 8.0, *p* = 0.001; electronic supplementary material, figure S2). Assigned families came from source patches with significantly lower *F*_ST_ values than what was expected from bootstrapping *F*_ST_ of available source patches per year (mean_[observed]_ = 0.08, mean_[expected]_ = 0.12, *p* = 0.001; electronic supplementary material, figure S2).

Of those assigned with a score of 100, 62% (*n =* 498) of the assignments were dispersal events. The proportion of assignments that were dispersal events versus non-dispersal events varied per year, from a low of 52% (*n =* 201) assigned as dispersal events in 2012 to a high of 78% (*n =* 190) in 2011. Mean dispersal distance ranged from 0.60 km in 2008 to 1.68 km in 2012, with an overall mean of 1.60 km, and the maximum distance recorded was 10.3 km (electronic supplementary material, figure S3).

### (b) Dispersal

**Figure 3.**
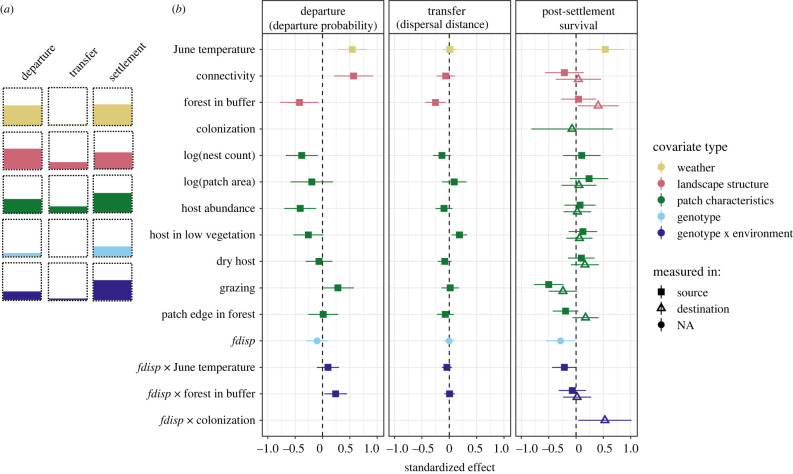
Effects of covariates on departure probability, dispersal distance and overwintering survival of dispersers. Points and whiskers in (*b*) are standardized coefficients and 95% confidence intervals from models presented in the electronic supplementary material, tables S1–S3. The shape of points indicates if variables were measured in source or destination patches. (*a*) Summarizes the largest observed effect of each covariate to the three stages of dispersal. (Online version in colour.)

**Figure 4.**
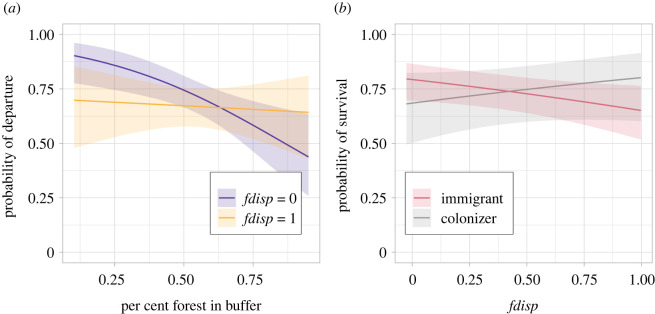
Genotype-by-environment interactions in departure and survival. Mothers that laid nests with higher *fdisp* were equally likely to disperse regardless of the surrounding forest matrix, whereas those with lower *fdisp* dispersed less when source patches were surrounded by a high amount of forest (*a*). Nests with higher *fdisp* were more likely to survive overwinter if they were the product of a colonization event rather than an immigration event (*b*). Lines show fitted predictions and 95% confidence intervals from the models presented in the electronic supplementary material, table S1 (*a*) and electronic supplementary material, S3 (*b*). For the purposes of presentation, we plotted predictions for the extremes of *fdisp* in (*a*). (Online version in colour.)

Departure probability was higher in warmer Junes, higher from source patches with high connectivity and lower from source patches with larger population sizes and more host plant and those surrounded by a higher percentage of forest matrix (figure 3; electronic supplementary material, table S1). Genotype alone was not associated with differences in departure probability; however, we found a significant genotype-by-environment interaction. Specifically, mothers of nests with lower frequencies of the dispersive allele (*fdisp*) were less likely to disperse when source patches were surrounded by a high amount of forest (figure 4*a*). Only two of our covariates showed significant associations with dispersal distance; dispersers moved shorter distances from patches surrounded by forest and longer distances from source patches that had more host plant growing in low vegetation (figure 3; electronic supplementary material, table S2). The mothers of nests with higher *fdisp* were not more likely to disperse longer distances.

Dispersers successfully settled in destination patches with significantly higher percentages of host growing in low vegetation, and patches with lower percentages of edge in forest compared to their source patch (electronic supplementary material, figure S4). Dispersers also settled in patches with significantly fewer nests compared to their source; however, this effect seemed to be driven by a high number of colonization events. After excluding colonization, we found a significant difference in the opposite direction; immigrants settled in destination patches that had significantly higher population sizes than their source patch (electronic supplementary material, figure S5).

### (c) Overwintering nest survival

We found that weather, landscape structure, the condition of source and destination patches, and genotype-by-environment interactions impacted overwintering survival of nests of dispersers. Nests of dispersers were more likely to survive over winter if (i) dispersal happened under warmer June conditions, (ii) destination patches were surrounded by higher percentages of forested landscape and (iii) source patches had lower levels of grazing (figure 3; electronic supplementary material, table S3). Nests with lower *fdisp* tended to have a higher chance of survival, especially when dispersal happened in warmer June conditions, but this interaction was not statistically significant. Nests with higher *fdisp* were significantly more likely to survive overwinter if they were the product of a colonization event compared to an immigration event with the opposite effect for nests with low *fdisp* (figure 4*b*). Dispersal distance did not influence survival—nests from long-distance dispersers had an equal probability of surviving overwinter as those from short-distance dispersers.

None of the patch characteristics associated with survival of dispersers were found to influence survival of residents; only the per cent of patch edge in forest had a significant positive effect on survival, and nests with higher frequencies of *fdisp* were more likely to survive overwinter (electronic supplementary material, table S4). Like dispersers, residents had higher chances of surviving overwinter when June temperatures were warmer; however, we found no evidence for genotype-by-environment interactions on survival for the residents (electronic supplementary material, table S4). Residents were slightly more likely to survive overwinter than dispersers, but this difference was not statistically significant (electronic supplementary material, table S5).

## 4. Discussion

### (a) Reconstructing dispersal

In total, we assigned 32% of our samples to a source patch, which translated to nearly 500 dispersal and 300 residency events. We found that on average butterflies dispersed 1.6 km and up to 10 km, which generally agrees with previously published estimates in this system from genetic studies [42] and harmonic radar [60], although is unsurprisingly higher than estimates from mark–release–recapture [55,61]. Our simulations suggest that we can expect reasonably low false positives in these assignments despite the complex structure of the metapopulation (electronic supplementary material, figure S1). Trans-generational assignment tests to reconstruct dispersal in butterflies is thus an encouraging approach, but clearly also one that benefitted from the massive effort to exhaustively genotype multiple individuals from all nests in all patches at large spatial scales. Feasibility was further enhanced because mating in this species occurs in the natal patch, increasing the likelihood that full-sibs of both parents were represented in allele frequencies of source patches. Although butterflies have long been a model organism for movement and dispersal ecology [62,63], to our knowledge our result is the largest reconstruction of realized dispersal for a butterfly in terms of spatial scale and the number of dispersal events captured.

However, it is important to consider that assignment tests come with inherent biases related to the underlying genetic structure of populations and the resolution of genetic markers [49,64,65]. In our case, assignments tended to be biased towards source patches with larger population size and families that had more genotyped larvae. Surprisingly, assigned source patches had significantly lower *F*_ST_ than expected (electronic supplementary material, figure S2). Twenty-eight per cent of nests were assigned to source patches that had an *F*_ST_ of less than 0.04; however; this matches well with the simulated data (24% of assignments had *F*_ST_ < 0.04 in the 50% sampled simulation) and many of these assignments were made to the same large source patches. Although simulated misassignment probability was inversely related to source patch *F*_ST_, misassignment probability remained low even when source patches were nearly undifferentiated from neighbours (electronic supplementary material, figure S1). Similarly low misassignments among populations with low genetic differentiation have been reported in other systems [66]. Our results must be interpreted considering these potential biases, recognizing that we are only capturing successful dispersal events, and we are likely disproportionately missing dispersal from the smallest of source populations and those that are highly connected.

### (b) Dispersal allows escape from poor-quality habitat

Dispersers tended to leave low-quality patches and settle in higher-quality patches. This suggests that environmental heterogeneity is an important driver of dispersal in this system, which is consistent with metapopulation theory [3,67]. Host plant abundance had the largest effect on departure, followed by grazing intensity and per cent of host growing in low vegetation (although the latter two were marginally non-significant; electronic supplementary material, table S1)—effects that are consistent in direction and magnitude with those predicting patch occupancy and abundance for the species in metapopulation models [53]. Dispersers were also more likely to leave and move further from patches containing few nests and settle in patches with many nests (after removing colonization events), confirming previous work that the species exhibits negative density-dependent dispersal [61,68]. Negative density-dependent dispersal is typically taken as evidence against kin competition. However, recent theoretical work found that an observation of low dispersal from large populations can result from a reduction in dispersal capacity in high-density sites due to reduced body condition from resource competition [69]. We found that dispersers moved longer distances out of patches with higher amounts of high-quality host plants (electronic supplementary material, table S2), which could suggest that dispersal is at least in part condition dependent. Although the species is not normally considered to be resource limited, larvae have been found to suffer from competition at very high densities [70]. Under experimental conditions, food-deprived *M. cinxia* larvae had lower flight metabolic rate as adults [71], and it is possible that food limitation in very large populations reduced emigration.

### (c) Dispersal propensity and dispersal distance are decoupled

While nearly all the environmental variables tested influenced the probability of departure, only few explained variation in dispersal distances. This suggests that the two processes could be under different constraints [1,15]. As expected, landscape structure influenced dispersal distances; dispersal distances tended to be longer from source patches surrounded by less forest (electronic supplementary material, table S2), suggesting that open landscapes are more permeable for *M. cinxia*. Unexpectedly, neither weather nor individual genotype influenced dispersal distances. While the probability of departure decreased in cooler summers, those that did disperse moved just as far as in warmer years. The lack of association between dispersal distance and genotype was especially surprising considering that females with higher *fdisp* fly further at moderate and low temperatures ([29,38,72], and nests with higher *fdisp* tend to be found at higher frequencies in newly colonized and isolated populations [72]—a pattern of spatial sorting that we also find in the current study among dispersers in isolated versus connected patches (electronic supplementary material, figure S6). Our results suggest that increased flight ability does not necessarily translate to increased realized dispersal distances, but that there is a spatial bias in dispersal paths among genotypes (figure 1). Nests with lower frequencies of the dispersive allele (*fdisp*) were more likely to be associated with dispersal events from source patches surrounded by less forest (figure 4). This indicates that females with low *fdisp* disperse just as far as those with high *fdisp* but tend to remain in areas of high permeability. Such a pattern might be expected if mobility is correlated with exploratory behaviour [73–76] or differential use of the landscape matrix [59,77]. However, as we capture only the endpoints of successful dispersal, it is possible that genotypes differ in their total flight distances or number of visited patches. Similarly, the observed mean dispersal distance of individuals with low *fdisp* might be overestimated if we are disproportionately missing dispersal assignments from well-connected patches.

### (d) Post-settlement costs of dispersal are indirect and hidden in genotype-by-environment interactions

Nests of dispersers versus residents (electronic supplementary material, table S5) and long-distance and short-distance dispersers (figure 3; electronic supplementary material, table S3) were equally likely to survive overwinter, suggesting that there is no direct cost to dispersal during the transfer phase among those that successfully dispersed [22]. This is consistent with previous experimental work that failed to find trade-offs between flight and reproduction [78,79] and even reported positive relationships between mobility and egg production [80]. Instead, some costs are apparently paid after settlement through direct negative effects of settling in poor-quality patches [81]. For example, we found that nests were less likely to survive overwinter if dispersers settled in patches with higher grazing. Although this effect was non-significant in the model (*p* = 0.07; electronic supplementary material, table S3), the effect of grazing was notably absent among residents (electronic supplementary material, table S4). Interestingly, the amount of grazing in the disperser's natal patch had much stronger effects on the survival of nests (figure 3; electronic supplementary material, table S3), suggesting that mothers from low-quality patches might decrease investment in reproduction at the cost of maintaining investment in flight. Effects of food stress on quality and quantity of offspring after flight have been found in other butterflies [82], including *M. cinxia*; Niitepõld [79] found no difference in flight metabolic rate in butterflies exposed to unlimited versus restricted food, but food-restricted females had significantly lower clutch size after flight.

Variation in post-settlement survival of the offspring among dispersing mothers might also explain how spatial sorting of genotypes can persist without differences in dispersal distances. We found no evidence of higher colonization (i.e. dispersal into an empty patch) probability among nests with higher *fdisp* (electronic supplementary material, table S6), but nests with higher *fdisp* were significantly more likely to survive overwinter if they were the product of a colonization event compared to nests with lower frequencies of *fdisp*. Those with high *fdisp* could thus have a selective advantage in the ability to establish in an empty patch. This could be related to the higher fecundity in *Pgi* heterozygote females ([78], who lay larger clutches even after long bouts of flight [78]). Group size is positively correlated with survival in experimental and natural conditions [83], and Hanski & Saccheri [84] found significantly higher growth rates of populations containing higher frequencies of *Pgi* heterozygotes after controlling for ecological factors, but only in small patches. It is less clear why nests with higher *fdisp* might be poor at establishing as immigrants, especially given that we found a positive association between *fdisp* and survival among residents (although note that positive trends are strong in only 2 of 4 years; electronic supplementary material, figure S7). This could be related to oviposition timing—females with the mobile *Pgi* genotype lay eggs earlier in the day compared to the less mobile genotype [85]. This would give those with high *fdisp* an advantage in accessing high-quality host plants in their natal patch, but as immigrants, this opportunity would be missed.

## 5. Conclusion

Our finding that different factors were important for departure, transfer and settlement highlights the multi-causality of dispersal and that effects are not easily generalizable from one stage to another. While extrinsic environmental factors played a stronger role in driving dispersal patterns, survival probabilities following dispersal crucially depended on genotype-by-environment interactions. Together our results suggest that if we are to accurately predict the impact of dispersal on population dynamics, an understanding of both the drivers of dispersal and deferred dispersal costs might be required.

## Data Availability

Data and scripts for the empirical analysis are available in the Dryad Digital Repository: https://doi.org/10.5061/dryad.7h44j0zws [86].
